# Patient Follow-up After Orthopaedic Outreach Trips – Do We Know Whether Patients are Improving?

**DOI:** 10.1007/s00268-022-06630-w

**Published:** 2022-06-28

**Authors:** Chelsea Leversedge, Samuel Castro, Luis Miguel Castro Appiani, Robin Kamal, Lauren Shapiro

**Affiliations:** 1 Stanford School of Medicine Department of Orthopaedic Surgery, VOICES Health Policy Research Center, 450 Broadway St, Redwood City, CA 94306 USA; 2 Stanford School of Medicine, 291 Campus Drive, Palo Alto, CA 94305 USA; 3 Department of Orthopaedic Surgery, Hospital Clinica Biblica Aveinda, 14 Calle 1 Y Central, San José, Costa Rica USA; 4 School of Medicine Department of Orthopaedics, University of California San Francisco, 1500 Owens Street, San Francisco, CA 94158 USA

## Abstract

**Background:**

The burden of traumatic musculoskeletal injuries falls greatest on low- and middle-income countries (LMICs). To help address this burden, organizations host over 6,000 outreach trips annually, 20% of which are orthopaedic. Monitoring post-surgical outcomes is critical to ensuring care quality; however, the implementation of such monitoring is unknown. The purpose of this review is to identify published follow-up practices of short-term orthopaedic surgery outreach trips to LMICs.

**Methods:**

We completed a systematic review of Pubmed, Web of Science, EMBASE, and ProQuest following PRISMA guidelines. Follow-up method, rate, duration, and types of outcomes measured along with barriers to follow-up were collected and reported.

**Results:**

The initial search yielded 1,452 articles, 18 of which were eligible. The mean follow-up time was 5.4 months (range: 15 days-7 years). The mean follow-up rate was 65.8% (range: 22%-100%), the weighted rate was 57.5%. Fifteen studies reported follow-up at or after 3 months while eight studies reported follow-up at or after 9 months. Fifteen studies reported follow-up in person, three reported follow-up via phone call or SMS. Outcome reporting varied among mortality, complications, and patient-reported outcomes. The majority (75%) outlined barriers to follow-up, most commonly noting transportation and costs of follow-up to the patient.

**Conclusions:**

There is minimal and heterogeneous public reporting of patient outcomes and follow-up after outreach trips to LMICs, limiting quality assessment and improvement. Future work should address the design and implementation of tools and guidelines to improve follow-up as well as outcome measurement to ensure provision of high-quality care.

**Supplementary Information:**

The online version contains supplementary material available at 10.1007/s00268-022-06630-w.

## Introduction

Approximately 5 billion people lack access to safe, affordable surgical care [1]. A large component of this burden stems from injuries and musculoskeletal disorders, which account for 16% of the world’s total burden of disease [2]. This injury burden disproportionately affects patients in low- and middle-income countries (LMIC), where nine in ten individuals cannot access basic surgical care [1]. To mitigate this gap in access, organizations across the USA sponsor over 6,000 outreach trips annually [3], 20% of which address orthopaedic surgical conditions [4]. While short-term surgical trips can improve access to surgical care, there is increasing concern regarding the lack of outcome collection and reporting among such trips [5, 6]. These trips commonly measure treatment success by outcome measures, such as the number of procedures performed as opposed to long-term patient outcomes or impact on local communities [5, 7, 8]. While current measurement systems often include utilization outcome measures (e.g. number of patients evaluated), these measures fail to capture quality of care, patient perspectives, or sustainability of the trip’s impact on the local healthcare system, which are foundational to safe and high-quality care [5, 8, 9].

Several organizations such as the Lancet Commission for Global Surgery and the G4 Alliance aim to address the lack of outcome collection during trips. Their guidelines for “safe and effective” surgery promote individual-level (e.g. access to healthcare) and system-level (e.g. adherence to protocols) data collection [1] alongside standardized measures (e.g. inpatient trauma mortality rate and surgical volume) [10]. Other surgical specialties are developing implementation tools to help collect outcomes after short-term surgical outreach trips [7, 11]. After reconstructive surgical outreach trips, for example, some organizations have connected patients with community health workers to reduce barriers such as distance and cost of travel [12]; others have adapted protocols to include telemedicine capabilities in order to increase follow-up and provide speech therapy [13]. Improving post-operative data collection is similarly important in orthopaedic surgery, yet little is little is known about follow-up practices following orthopaedic surgical outreach.

To identify the current follow-up practices during orthopaedic surgical outreach trips, we completed a systematic review to analyse (1) what are the reported follow-up practices following orthopaedic outreach trips, (2) what outcomes are collected, and (3) what are the barriers to conducting follow-up during such trips?

## Material and Methods

### Search Strategy and Selection Criteria

**Fig. 1:**
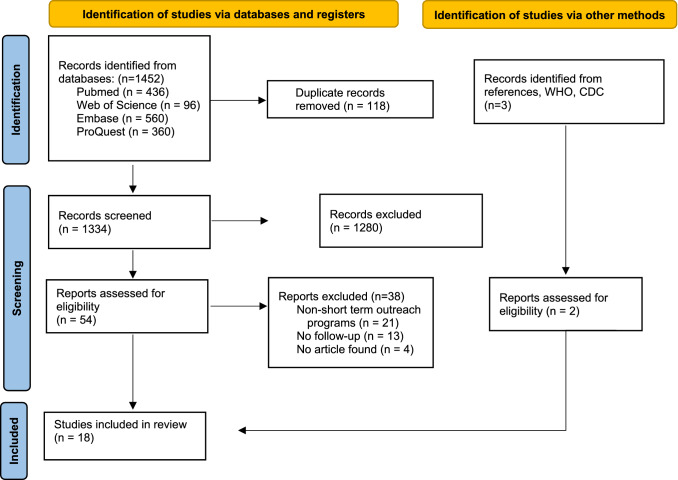
PRISMA Diagram of Search Strategy and Results Adapted from: Page MJ, McKenzie JE, Bossuyt PM, Boutron I, Hoffmann TC, Mulrow CD, et al. The PRISMA 2020 statement: an updated guideline for reporting systematic reviews. BMJ 2021;372:n71. https://doi.org/10.1136/bmj.n71. For more information, visit: http://www.prisma-statement.org/

We conducted a systematic review to identify all literature including patient follow-up after orthopaedic outreach trips. We followed the Preferred Reporting Items for Systematic Reviews and Meta-Analyses (PRISMA) guidelines (Fig. 1) [9]. This review was registered with Prospero (https://www.crd.york.ac.uk/prospero/), an international prospective register of systematic reviews before data extraction began (Prospero ID: 291,575). Explicit search algorithms were designed with the assistance of two research librarians to search Pubmed, Web of Science, EMBASE, and ProQuest. The initial search was for all studies filed under the search terms of “outreach missions”, “orthopaedics”, and “follow-up”, with appropriate MeSH Terms and synonyms for each. The search was limited to peer-reviewed articles focusing on orthopaedic outreach trips (or trips including orthopaedic surgeries and providers), published in English after 1990. All study types, except for systematic reviews, case studies, and perspective papers, were eligible for inclusion. Other exclusion criteria included non-orthopaedic outreach trips and long-term outreach or partnerships (such as global health surgical centres or non-governmental organization (NGO)-supported local programmes).

Following the database searches, results were uploaded onto Covidence, a Cochrane-sanctioned application for screening and analysing of articles in systematic reviews. Two authors independently conducted the initial screening (CL and SC), which included the screening of titles and abstracts, before discussing discrepancies during a research meeting. Two authors (CL and SC) then conducted a full-text review and reviewed the references of each included article and the grey literature to identify missing literature.

### Quality Assessment and Data Collection

After screening, we performed a quality assessment of the eligible articles using the Newcastle–Ottawa Scale (NOS) for cohort studies. The NOS assesses 3 domains: selection of participants, comparability of the groups, and quality of the outcomes [14]. A higher score (ranging from 0–9) indicates a higher-quality study, with scores < 7 indicating a low-quality study that will not be included in the analysis. For case series studies, we used the IHE Quality Appraisal Tool [15, 16]. Developed through the Delphi technique, this tool includes 18 questions and assesses potential bias in case series studies [16].

### Data Extraction and Analysis

Two authors (CL and SC) extracted article information onto a shared spreadsheet. Initial extraction included article information such as study type (case series, cohort, etc.) along with basic characteristics of the outreach trip. These included geographic location of the trip, range of data collection, patient population (adult, paediatric, or mixed), and the surgical specialty focused on during the trip (e.g. hand, spine, general).

Following guidelines from other systematic reviews examining follow-up practices, we extracted follow-up rate, length of follow-up, and health outcomes [5]. Length of follow-up was defined as the time between surgical care and the follow-up event. It was reported either as noted in the studies or as a mean for studies indicating time ranges or multiple follow-up appointments (e.g. patients returning for follow-up between 1–2 years was averaged as 1.5 years) [17]. Follow-up rate was defined as the number of patients who returned at the specified date or time for a follow-up appointment divided by the total number of patients receiving care during the outreach trip. For studies reporting multiple time points of follow-up, each point was extracted separately and then averaged to capture minimum and maximum follow-up. We then calculated a weighted average for patient follow-up by number of patients who received surgical care within each study to analyse any potential confounding of the mean by sample size. We also collected the platform used for follow-up (technology-based or in person).

We extracted outcomes collected by each trip or organization, including mortality, complications, complication rate, and patient-reported outcomes (PROs). We collected the complications reported and complication rates, reported as the per cent of all patients operated on. From studies reporting multiple complication rates, we averaged the rates, and then calculated the overall mean complication rate. We extracted three components of PROs: the patient-reported outcome measure(s) (PROM(s)) utilized, PROM use time points (e.g. pre-operatively, during follow-up), and any modifications to the tool (e.g. translation, adaptation).

We collected and categorized barriers to follow-up using a socioecological framework, which categorizes factors based upon the level at which they impact health—individual, community, organizational, and social—and the interaction between the levels [18–20]. Within this framework, the individual level includes personal characteristics, such as education, income, and knowledge [20]. The second category either includes relationships or organizations, such as organizational resources or policies [19]. The third category is community, which includes an individual’s setting or environment [20], and the fourth outlines social influences such as policies and broad societal factors (e.g. culture or social norms) [18, 20]. The socioecological framework has recently been used to evaluate various subspecialties including orthopaedic surgery [18, 21] and is often used to evaluate barriers to follow-up [22, 23]. By mapping barriers to each level, implementation strategies can be applied to ensure that barriers at all levels are addressed during programming [18]. Within this study, barriers to follow-up were mapped to the four levels.

As cost-effectiveness is often reported as a measurement of outreach trip sustainability [5, 8, 24], we extracted cost and cost-effectiveness data reported from outreach trips and, more specifically, from follow-up conduction.

## Results

The initial search yielded 1452 articles; 118 duplicates were removed. Eighteen articles were included (Fig. 1). All included articles met quality analysis thresholds. Table 1 demonstrates the details of included studies.

**Table 1:** Included Studies

Study	Outreach location	Trip focus	Number of patients treated	Per cent of patients returned for follow-up	Length of follow-up	Method of follow-up
Armstrong et al.[27]	Vavuniya, Sri Lanka	Spinal cord injuries	89	31.5%	6–12 weeks	Telephone calls
Bido et al. [17]	Dominican Republic	Total joint	194	80%	1–2 years	In person (host provider)
Chuang et al. [33]	Honduras	Hand surgery	63	82%	4 months	In person (host provider)
Cousins et al. [39]	Kenya	General Orthopaedics	187	77%	12 weeks	In person, host and visiting provider
Dempsey et al. [32]	Dominican Republic	General Orthopaedics	58	79%	1 year	In person (host provider)
Doman et al. [37]	Honduras	Hand surgery	57	100%	15 days	In person, host and visiting provider
Hu et al., [28]	Brazil	Total hip arthroplasty	38	87% at 2 weeks, 84% at 6 weeks, 66% at 12 weeks, 29% at 1 year	2, 6, 12 weeks, and 1 year	Telephone call
Irmay et al. [30]	Sierra Leone	Krunkenberg procedure	11	90.1%	3 months	In person (host provider)
Pigeolet et al. [34]	Bangladesh	Clubfoot	22	22% at 3 months, 28% at 9 months, 28% at 14 months, 84% at 24 months At least 90.6% attended 1 appointment	3, 9, 14, and 24 months	House visits (host–physician)
Raissi et al. [38]	Iran	Spinal cord	122	50%	8 months	In person (by visiting provider)
Schlegelmilch et al., [35]	Ecuador	Total hip arthroplasty	157	58.6% at 1 year	6 weeks, 3mo, 6mo, and 1 year	In person, host provider at 6 weeks, 3 months, and 6 months, with visiting provider at 1 year
Shapiro et al. [48]	Vietnam	Hand surgery	8	87.5% at Day 1, 87.5% at Week 1, 100% at Week 2, 75% at Week 4, 100% at week 12	12 weeks (Day 0, Day 1, Week 1, Week 2, Week 4, Week 12)	SMS messaging
Stenquist et al. [36]	Dominican Republic	Total knee arthroplasty	192	41%	1–4 years	In person, host and visiting medical student
Teicher et al. [31]	Haiti	Trauma, orthopaedic cases	248	42% (at 45th day) “Majority” present for 3–6-mo follow-up appointment	45 days and 3-6 months	In person (host provider)
Torchia et al. [40]	Peru	Predominantly Orthopaedic trauma	127	81.90%	2–4 weeks, 5–7 weeks, 4–7 months, 8–12 months	In person (at local clinic or home visits)
Walk et al. [41]	Central & South America (US Naval ship)	Paediatric surgery (including orthopaedics)	340	NS	NS	In person, database shared with visiting physicians
White et al. [25]	Benin	Orthopaedics, plastics, and maxillofacial	545 (346 invited for follow-up)	43% of those invited	4–10 months, median of 8 months	In person (returning visiting providers for evaluation day)
White et al. [26]	Sub-Saharan Africa	Orthopaedics, plastics, and maxillofacial	641 (174 invited for follow-up)	40.8% of those invited	7 years	In person (returning visiting providers for evaluation day)

**Fig. 2:**
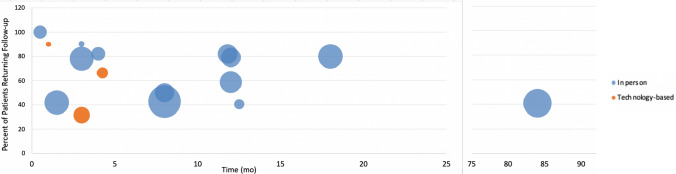
Patient follow-up rate over time Each study’s reported follow-up method is represented by a colour (via technology (orange) or in person (blue)) and total number of patients seen during the trip(s) is represented by the size of each datapoint

A total of 2,759 patients received care across all included orthopaedic outreach trips, with 2,093 (75.8%) offered at least one follow-up opportunity. Three studies (16.7%) did not offer follow-up to all patients, two of which (11%) invited a select percentage of patients due to time and logistical constraints [25, 26] and one transferred a patient to another hospital [27]. The mean length of all follow-up events was 5.4 months and the mean maximum follow-up length offered was 6.5 months. All studies conducted follow-up for at least 15 days post-operatively. The number of studies collecting follow-up decreased as time increased; fifteen (88%) studies reported follow-up at or after 3 months and eight (47%) reported follow-up at or after nine months. The average follow-up rate was 65.8%, ranging from 22%-100% attendance at follow-up (Fig. 2). The weighted follow-up rate was 57.5%. The majority reported in-person follow-up (83%) while three (16.7%) used technology-based platforms (SMS or phone calls) [27–29].

Two studies specifically reported mortality rates [30, 31], in one, all patients survived [30], in another study, six (2.4%) patients died [31]. Twelve studies (61%) reported complications; however, there was heterogeneity in complication reporting. Seven of those studies reporting complications outlined complication rates by complication type, two studies reported the number of patients “doing well” at follow-up [17, 32] (Table 2). For studies reporting complications, the average rate of complications was 18.3%. PRO collection was reported in eleven (61%) studies; nine used PROs [17, 27, 29, 33–36], one collected PROs via semi-structured interviews [25], and one used a combination [26]. Of those using PROs, two did not specify any tool modifications [27, 28], six specified using translated tools [9, 13, 15, 16, 37], and two included a translated and culturally adapted tool [29, 34]. The WOMAC (Western Ontario and McMaster Universities Osteoarthritis Index) and Quick-Disabilities of the Arm, Shoulder, and Hand (QuickDASH) were the only two instruments measuring patient-reported function and physical health utilized in more than one study. The SF-36 mental health subscale was the most commonly utilized instrument to collect data related to mental health [17, 32, 33, 35].

**Table 2:** Outcome collection during follow-up

Study	Patient-Reported Outcome	PROM use time points	Complication rate	Measured complications
Armstrong et al. [27]	Spinal Cord Independence Measure II (SCIM); American Spinal Injury Association Impairment Scale (ASIA)	Pre-op, post-op, and at follow-up	22.6% at discharge, 7.9% readmission, pressure ulcers: 33%, UTI: 5.6%, Bowel problems: 6.7%, Pain: 36.0%, Psychological problems: 10.1%	Presence of complications (broken down by pressure ulcers, UTIs, bowel problems, readmission to rehabilitation, pain, and psychological problems) *note: data only available for 51 of the 89 treated patients
Bido et al. [17]	(1) WOMAC and (2) SF-36 mental health subscale	Pre-op and follow-up	NS	Perceived chance of complications, change in pain
Chuang et al. [33]	(1) QuickDASH, (2) SF12v2 (abbreviated SF-36), and (3) Satisfaction Survey	Pre-op, post-op, and at follow-up	NS	Satisfaction rating with pain level
Cousins et al. [39]	NS	NS	15.5%	Presence of complications, compared to reports of “doing well”
Dempsey et al. [32]	(1) WOMAC and (2) SF-36	Pre-op and Follow-up	NS	Perceived chance of complications
Doman et al., [37]	NS	NS	5.4%	Presence of complications
Hu et al. [28]	(1) Harris Hip Score, (2) PROIS-SF (Patient-Reported Outcome Measurement Information System Short-Form), and (3) PROIS-SF Physical Function	Pre-op and Follow-up	13.2%	Presence of complications
Irmay et al., [30]	NS	NS	36.4%	Complications requiring a second procedure
Pigeolet et al., [34]	(1) International Clubfoot Study Group Outcome evaluation score, (2) Laaveg–Ponseti score, and (3) a social questionnaire	Follow-up survey	4.5%	Decreased quality of life post-operatively
Raissi et al. [38]	NS	NS	96.3% burning pain, 9.3% bladder problems, 35.2% pressure ulcers	Presence of complications (pain, UTIs and bladder problems, and pressure ulcers)
Schlegelmilch et al. [35]	SF-36 or 15D	Follow-up survey	NS	NS
Shapiro et al. [48]	QuickDASH	Follow-up survey	NS	NS
Stenquist et al. [36]	Semi-structured interviews on patient satisfaction, return to activity, and mental health	Follow-up patient interviews	NS	NS
Teicher et al. [31]	NS	NS	3.2%	Infection rate and good callous (satisfactory bone healing)
Torchia et al. [40]	NS	NS	7.9%	Presence of complications (broken down by infection rate, malunion or non-union, nerve injuries, and complications requiring a second surgery)
Walk et al. [41]	NS	NS	1.2%	Presence of complications
White et al. [25]	WHODAS 2.0 (World Health Organization Disability Assessment Schedule 2.0), including 6 point Smiley Face Assessment Scale	Follow-up interviews and surveys	5% of all patients, 21% of those who returned for f/u	Residual pain
White et al. [26]	Semi-structured interviews on patient satisfaction, social impact and patient’s perception of care received vs. expectations. Patient pain measured with Wong and Baker faces scale (paediatric population) and 6 point Smiley Face Assessment Scale (adults)	Follow-up patient interviews	5.9% of all patients, 62% of those who returned for f/u	Presence of complications (broken down by seeking further treatment, slow healing, wound breakdown, and residual pain)

**Fig. 3:**
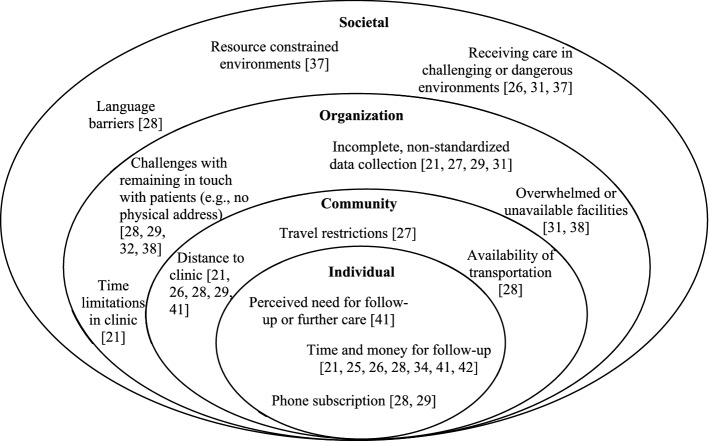
Noted barriers to conducting follow-up during short-term orthopaedic outreach trips are displayed by level of the socioecological framework

Barriers to follow-up were identified in fourteen (78%) studies and were found in each of the four levels of the socioecological framework (Fig. 3) [18, 21, 25–29, 31, 32, 34, 37–41]. The organization level had the greatest number of barriers (e.g. overwhelmed facilities [31] or a lack of a patient database [42]). However, the most commonly identified barrier was cost of follow-up to patients, both monetary and time costs, identified in six (22%) studies [25, 26, 28, 39–41]. Cost was primarily reported as it relates to financial assistance for patients (e.g. payment for transportation) [25–28, 34, 35, 39, 40]. Reported cost information is illustrated in Supplementary Table 1.

## Discussion

Outreach trips to LMICs are increasing to meet the burden of musculoskeletal disease and trauma, but outcome evaluation and patient follow-up are lacking after most trips. This systematic review illustrates current follow-up practices after orthopaedic outreach trips, demonstrating heterogeneity in follow-up methods and sparse outcome reporting, and informs areas for improvement efforts.

The results of this systematic review should be viewed in light of their limitations. This review predominantly includes case series, with few studies including a measured intervention. Overall, the number of eligible studies was limited, representing a small number of the estimated thousands of orthopaedic outreach trips conducted annually [3, 4]. It is important to note that long-term programmes, such as skills transfer programmes, were not included. Additionally, organizations may collect data internally without resources or academic partners to support publishing externally. We similarly recognize that it is not necessary for all trips to report and publish follow-up findings [43]. Nonetheless, this review may introduce bias as organizations more likely to purposefully collect data may be over-represented here. The mean follow-up rate and duration in papers included in this review are likely greater than the follow-up rate and duration occurring after trips that do not publish data. The lack of robust data, both in quantity of studies and heterogeneity of reporting outcomes, hindered the ability to run statistical analyses on the data. Despite these concerns, the quality of studies was good based on respective quality assessment scores (> 77% on NOS, > 70% on IHE) [14–16]. Furthermore, the rate and duration of follow-up in high-income countries (HICs) is not known with certainty and varies by many factors (e.g. patient population, location), which limits comparison.

We analysed unweighted and weighted follow-up rate means and noticed a discrepancy. The fact that the weighted mean (57%) is lower than the non-weighted mean (66%) suggests that studies with high follow-up rates and smaller sample sizes influenced the unweighted mean. The lower rate of follow-up on greater sample sizes has implications for follow-up feasibility. Both means, however, are similar to those reported of surgical outreach trips conducted by other specialties. For example, a systematic review of reconstructive outreach trips found an average 56% return for follow-up [5], and another reported a 14–84% range [8].

Of the two forms of follow-up, follow-up rates were higher in studies using technology-based methods (SMS and telephone calls) than during in-person follow-up. Although previous studies indicate that technology-based follow-up can pose challenges (e.g. physical examination difficulties) [44], studies in both HIC and LMIC have highlighted time and cost savings to patients which may help address barriers to follow-up [45–47]. Technology-based follow-up might also improve sustainability, as the return or longer stay of visiting surgeons may not be sustainable, nor does it promote local surgical capacity. White et al. noted this decreased sustainability, as logistical and time constraints limited the number of patients visiting providers were able to invite for follow-up when revisiting the site seven years post-operatively [25]. In the future, focusing on technology-based and host–physician-led follow-up may promote more sustainable partnerships, build capacity, and improve the feasibility of follow-up [5, 13, 48].

Irrespective of reported follow-up method, outcomes reporting was limited and heterogeneous across included studies. Only about half of the studies reported complications, which makes drawing conclusions regarding trip effectiveness challenging [5, 8, 49] and does not allow for comparison across different outreach trips, which can decrease accountability [49]. Among those reporting complications, the average complication rate was 18.3%, which is concerning in comparison to the global orthopaedic complication rate of 5% [50]. In the USA, for example, complication rates are less than 2% following open reduction and internal fixation (ORIF) of ankle fractures [51] and approximately 8% following orthopaedic trauma surgery [52]. In this review, only three studies [31, 39, 40], all of which primarily treated traumatic injuries, specifically reported infection rates. The average infection rate of 2% among all patients who received follow-up in these three studies is much lower than that reported in other studies examining infection rates after similar procedures. For example, post-surgical infection rates are higher following traumatic injuries treated by ORIF in LMICs (6.4%) [53] and following orthopaedic surgery in the USA (about 4%) [54, 55], perhaps representing a lack of follow-up and/or under-reporting within the studies in this review [5, 54, 56]. The discrepancies in these numbers and the high complication rates highlight how outreach organizations have a responsibility to conduct follow-up or ensure follow-up is attainable.

In contrast, many studies reported PRO collection in an effort to capture patient perspectives. It should be noted, however, the application of PROs in these settings is limited. Only two studies reported tool adaptation to the community context, an approach known as community translation [29, 34] or the adaptation of tools by the target population to fit the needs and livelihoods of the population addressed, beyond translation [57, 58]. This adaptation captures cultural and linguistic differences between populations (for example, QuickDASH includes golf and tennis, which may not be globally relevant). Cultural differences were noted as a barrier to follow-up within this review [26, 35], mirrored in other studies showing how a lack of cultural adaptation results in higher attrition rates [57]. Additionally, tailoring tools to the patient population promotes shared decision-making and patient-centred care by building trust and incorporating patients’ values [42, 58–60].

Barriers are similar to those noted in prior studies [9, 13, 47] and can be viewed as they relate to the socioecological framework, which allows for identification of improvement opportunities within and across levels in a systematic manner. Four studies utilized follow-up methods that address multiple barriers at once, which, as a result, addresses multiple levels of the socioecological framework. For example, the use of SMS message-based follow-up addresses time for follow-up (individual level), distance to follow-up (community level), and incomplete outcome data collection (organization) [29]. Such data can be integrated back into electronic health record systems for patient monitoring [61]. Each study that addressed multiple levels of the socio-economic framework had higher-than-average rates of follow-up [28, 29, 32, 40].

The lack of and barriers to follow-up after orthopaedic outreach trips present a cause for concern and raise questions regarding ethical challenges of providing orthopaedic care in LMIC. Local care professionals and patients treated during outreach also share this concern, as studies examining outreach experience demonstrate a desire for longer follow-up [62–64]. Pean and colleagues developed an ethical framework for global orthopaedic surgery that details the importance of “implementing periodic follow-up and identifying and addressing complications”, yet the implementation of this framework has not been described [65]. Moving forward, the development and implementation of global guidelines, quality measures, and sustainable tools for follow-up is vital [47, 66]. These changes are necessary to hold orthopaedic outreach organizations accountable and to ensure the provision of high-quality care.

## Conclusion

As the number of orthopaedic outreach trips to LMICs continues, it is important to ensure that patients are followed post-operatively to verify care delivery is safe and of high quality. This review not only demonstrates the paucity and heterogeneity of follow-up after orthopaedic outreach trips but also illustrates the need for further investigation and potential opportunities to ensure accountability and the provision of high-quality care. Future work in exploring technology-based follow-up and in guidelines for the duration, type, and metrics of follow-up after orthopaedic outreach trips may be useful moving forward.

## Supplementary Information

Below is the link to the electronic supplementary material.

Supplementary file1 (DOCX 14 KB)
