# Supplementary material for: Patient Follow-up After Orthopaedic Outreach Trips – Do We Know Whether Patients are Improving?
Source: World J Surg. 2022 Jun 28;46(10):2299–309. doi: 10.1007/s00268-022-06630-w (PMC9436850; doi:10.1007/s00268-022-06630-w)
Supplement: Supplementary file 1 — Supplementary file1 (DOCX 14 KB) [file 268_2022_6630_MOESM1_ESM.docx]

Supplementary Table. Noted Trip Costs and Associated Details

|  | **Costs to patients and local providers** | **Methods in place to decrease costs** | **Cost Breakdown** |
| --- | --- | --- | --- |
| Armstrong et al., 2014 | NS | NS | Total cost of trip: €664,818  67% of all trip costs: related to trip staffing and length of stay |
| Cousins et al., 2012 | Cost burden of surgery and of follow-up for patients | NS | NS |
| Doman et al., 2011 | Limit on ability to bring supplies based on cost | NS | NS |
| Pigeolet et al., 2022 | Cost burden of follow-up | Financial support offered to cover transportation costs | NS |
| Schlegelmilch et al., 2017 | NS | NS | Cost-effectiveness ratios:  $4,442 for unilateral hip arthroplasty (HA), $2,939 for bilateral HA,  $4,392 for staged procedures  67% of total trip cost: related to trip staffing and length of stay |
| Torchia et al., 2016 | Cost burden of follow-up | No charge to the patient for follow-up and reduced cost for follow-up radiographs (USD $5).  Reimbursement model for local providers based on number of follow-up events (e.g. follow-up within 2-4 weeks = base pay, within 8-12 months = 5x base pay) | Annual cost of follow-up program: $20,041 |
| White et al., 2017 | Cost burden of follow-up | Limited number of patients invited to follow-up | NS |
| White et al., 2018 | Cost burden of follow-up | Limited number of patients invited to follow-up | NS |
